# Androgen deprivation therapy sensitizes prostate cancer cells to T-cell killing through androgen receptor dependent modulation of the apoptotic pathway

**DOI:** 10.18632/oncotarget.2429

**Published:** 2014-09-03

**Authors:** Andressa Ardiani, Sofia R. Gameiro, Anna R. Kwilas, Renee N. Donahue, James W. Hodge

**Affiliations:** ^1^ Laboratory of Tumor Immunology and Biology, Center for Cancer Research, National Cancer Institute, National Institutes of Health, Bethesda, MD, USA

**Keywords:** enzalutamide, abiraterone, ADT, cancer vaccine, immunogenic modulation, prostate cancer, immunotherapy

## Abstract

Despite recent advances in diagnosis and management, prostrate cancer remains the second most common cause of death from cancer in American men, after lung cancer. Failure of chemotherapies and hormone-deprivation therapies is the major cause of death in patients with castration-resistant prostate cancer (CRPC). Currently, the androgen inhibitors enzalutamide and abiraterone are approved for treatment of metastatic CRPC. Here we show for the first time that both enzalutamide and abiraterone render prostate tumor cells more sensitive to T cell-mediated lysis through immunogenic modulation, and that these immunomodulatory activities are androgen receptor (AR)-dependent. In studies reported here, the NAIP gene was significantly down-regulated in human prostate tumor cells treated *in vitro* and *in vivo* with enzalutamide. Functional analysis revealed that NAIP played a critical role in inducing CTL sensitivity. Amplification of AR is a major mechanism of resistance to androgen-deprivation therapy (ADT). Here, we show that enzalutamide enhances sensitivity to immune-mediated killing of prostate tumor cells that overexpress AR. The immunomodulatory properties of enzalutamide and abiraterone provide a rationale for their use in combination with immunotherapeutic agents in CRPC, especially for patients with minimal response to enzalutamide or abiraterone alone, or for patients who have developed resistance to ADT.

## INTRODUCTION

Androgen deprivation therapy (ADT) is a standard of care for prostate cancer [1, 2]. However, most patients eventually develop castration-resistant prostate cancer (CRPC). CRPC was generally thought to be completely resistant to ADT. However, it has been demonstrated that CRPC remains dependent on androgen signaling for growth and that CRPC is sensitive to further manipulation of androgen signaling [3]. Two agents FDA-approved for ADT currently play a major role in the management of CRPC: enzalutamide and abiraterone. Enzalutamide is an androgen receptor (AR) antagonist that blocks androgens from binding to the AR and prevents nuclear translocation and coactivator recruitment of the ligand-receptor complex. The utility of enzalutamide has been demonstrated in clinical trials [4-6], including the AFFIRM trial where it mediated a 4.8-month advantage in overall survival compared to placebo [6]. Abiraterone is a potent inhibitor of CYP17A1, a rate-limiting enzyme in androgen biosynthesis. Inhibition of this enzyme subsequently blocks the production of androgen in all endocrine organs, including the testes, adrenal glands, and in the prostate tumor itself [7]. In a phase III study in patients with CRPC previously treated with docetaxel, abiraterone was shown to improve overall survival by 3.9 months compared to placebo [8].

Immunogenic modulation, whereby conventional therapies alter tumor phenotype, rendering tumor cells more susceptible to immune-mediated attack, has been described previously and reviewed [9]. The immunomodulatory effects of conventional therapies such as chemotherapy or radiation include up-regulation of tumor antigens, Fas, MHC moieties, and components of the antigen-processing machinery (APM). The various immunomodulatory effects of standard therapies can be exploited to enhance the antitumor activity induced by immunotherapy. The immunogenic modulation potential of ADT, particularly enzalutamide, was first described in a mouse model. Treating murine prostate carcinomas with enzalutamide *in vitro* increased expression of MHC-I and Fas on the cell surface, which subsequently improved the sensitivity of TRAMP-C2 cells to T cell-mediated killing [10]. The ability of enzalutamide to sensitize tumor cells to immune-mediated killing enhanced the efficacy of combination treatment with enzalutamide and a therapeutic cancer vaccine, which translated to significant improvement in overall survival of TRAMP mice (27.5 vs. 10.3 weeks) compared to ADT or vaccine therapy alone.

Here, we investigated whether ADT mediated immunogenic modulation and rendered human prostate carcinomas more sensitive to T cell-mediated killing. To our knowledge, this is the first study to report a) the novel immunomodulatory properties of ADT with enzalutamide or abiraterone that render human prostate carcinomas more sensitive to immune-mediated attack; b) that the immunogenic modulation properties of ADT are dependent on AR expression; c) that the molecular mechanism of enzalutamide-mediated immunogenic modulation in human prostate carcinomas includes modulation of the expression of the antiapoptotic gene NAIP (NLR family, neuronal apoptosis inhibitory protein); d) the functional importance of NAIP in rendering human prostate tumor cells sensitive to immune-mediated killing; and e) that enzalutamide renders prostate tumor cells harboring AR amplification (the major mechanism of ADT resistance) more sensitive to T-cell mediated killing. These data further support the combination of ADT and immunotherapy as a promising treatment for CRPC.

## RESULTS

### ADT with enzalutamide or abiraterone inhibited proliferation of AR^+^ prostate tumor cells and increased their sensitivity to T-cell killing

**Figure 1:**
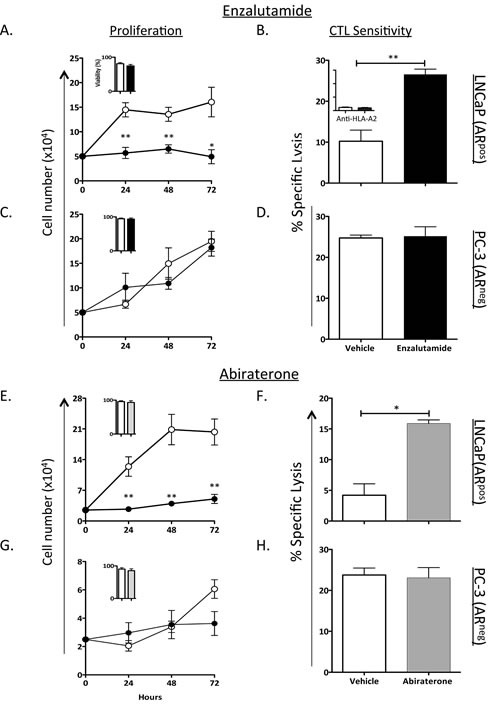
ADT inhibited the growth of AR^+^ prostate tumor cells and improved their sensitivity to T cell-mediated killing The human prostate tumor cell lines LNCaP (AR^+^; HLA-A2) (A) and PC-3 (AR^−^, HLA-A24) (C) were treated with vehicle (DMSO; open symbols) or 10 μM enzalutamide (closed symbols). Cell proliferation was determined at indicated time points. After 48 h of either vehicle or enzalutamide treatment, LNCaP (B) and PC-3 (D) cells were used as targets in a CTL lysis assay using MUC1-specific CD8^+^ T cells as effector cells at an E:T ratio of 30:1. (B) Inset, LNCaP cells treated with vehicle or enzalutamide were used as CTL targets in the presence of an anti-HLA-A2 blocking antibody. LNCaP and PC-3 cells were treated with vehicle (open circles) or 10 μM abiraterone (closed circles) (E and G respectively). (E-H) The effect of abiraterone on LNCaP (E-F) and PC-3 (G-H) cell proliferation and CTL sensitivity to MUC1-specific CD8^+^ T cells as effector cells was determined. Viability of the cells tested was assessed at 72 h after treatment by trypan blue exclusion (insets). Results are presented as mean ± S.E.M. from 3–6 replicate wells. Asterisks denote statistical significance relative to controls (*P < 0.05, **P < 0.01). These experiments were repeated 3–5 times with similar results.

Enzalutamide has previously been shown to induce immunogenic modulation in TRAMP-C2 mouse prostate carcinomas and to improve tumor cells' sensitivity to gp70-specific cytotoxic T-lymphocyte (CTL) killing *in vitro* [10]. Here we investigated the effect of ADT with enzalutamide or abiraterone on human prostate carcinomas. To determine the effect of ADT on tumor-cell proliferation, 2 human prostate tumor-cell lines, LNCaP (AR^+^, HLA-A2) and PC-3 (AR^−^, HLA-A24), were treated *in vitro* with vehicle (DMSO) or 10 μM enzalutamide or abiraterone. This clinically relevant dose was similar to or lower than the median plasma concentration achieved in humans [11]. Treatment with enzalutamide significantly inhibited the growth of LNCaP cells (*P* < 0.01) (Fig. 1A), but did not inhibit the proliferation of PC-3 cells (Fig. 1C). Similarly, abiraterone significantly reduced the proliferation of LNCaP cells (*P* < 0.01), but did not affect PC-3 cells (Figs. 1E and 1G). Neither enzalutamide nor abiraterone affected the viability of LNCaP and PC-3 cells, as measured by trypan blue exclusion after 3 days of drug exposure (insets, Figs. 1A, 1C, 1E, and 1G). To determine whether enzalutamide or abiraterone mediated increased sensitivity to T-cell lysis, LNCaP and PC-3 cells were treated with either drug *in vitro* and used as target cells for MUC1-specific CTL-mediated killing assays. Exposing LNCaP cells to enzalutamide significantly enhanced their sensitivity to MUC1-specific CTL-mediated lysis relative to tumor cells exposed to vehicle (*P* < 0.01) (Fig. 1B). This killing was MHC-restricted as determined by HLA-A2 blocking (Fig. 1B inset). Similarly, exposing LNCaP cells to abiraterone significantly improved their sensitivity to MUC1-specific CTL-mediated lysis compared to vehicle-treated tumor cells (*P* < 0.05) (Fig. 1F). However, neither enzalutamide nor abiraterone improved PC-3 cells' sensitivity to MUC1-specific CTL-mediated lysis (Figs. 1D and 1H) relative to vehicle-treated tumor cells. These results suggested that both enzalutamide and abiraterone mediated immunogenic modulation in human prostate tumor cells, and this effect was dependent on AR expression.

### Immunogenic modulation by enzalutamide was dependent on AR expression

**Figure 2:**
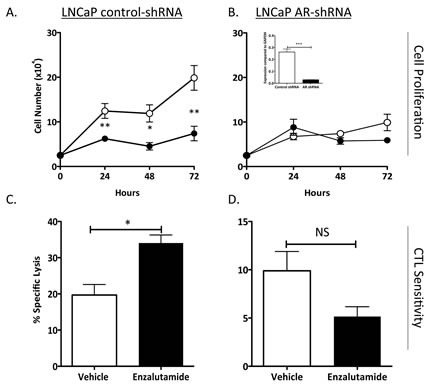
Increased CTL sensitivity by enzalutamide was dependent on AR expression Human prostate tumor cell lines LNCaP expressing control-shRNA (AR^+^, HLA-A2) (A) and LNCaP AR-shRNA (AR^−^, HLA-A2) (B) were treated with vehicle (DMSO; open symbols) or 10 μM enzalutamide (closed symbols). Cell proliferation was determined at indicated time points. AR expression levels were confirmed by RT-PCR (inset). After 48 h of either vehicle or enzalutamide treatment, LNCaP control-shRNA (C) and LNCaP AR-shRNA (D) cells were used as targets in a CTL lysis assay using CEA-specific CD8^+^ T cells as effector cells at an E:T ratio of 30:1. Results are presented as mean ± S.E.M. from 3–6 replicate wells. Asterisks denote statistical significance relative to controls (*P < 0.05, **P < 0.01, ***P < 0.001 (inset), NS: not significant). These experiments were repeated 3–5 times with similar results.

To further confirm that immunogenic modulation by enzalutamide is AR-dependent, we used a pair of LNCaP cell lines stably expressing either control-shRNA (expresses AR) or AR-shRNA cells (reduced or no AR expression) [12]. *In vitro*, enzalutamide significantly inhibited the proliferation of LNCaP control-shRNA (*P* < 0.01) (Fig. 2A) but did not affect the growth of LNCaP AR-shRNA (Fig. 2B). Treatment with enzalutamide significantly enhanced the sensitivity of LNCaP control-shRNA to carcinoembryonic antigen (CEA)-specific CTL-mediated lysis compared to vehicle-treated tumor cells (*P* < 0.05) (Fig. 1C). The improved sensitivity to T-cell killing mediated by enzalutamide was lost when tumors with reduced expression of AR were used, as seen in Figure 2D, where LNCaP AR-shRNA treated with enzalutamide or vehicle demonstrated similar sensitivity to CEA-specific CTL-mediated lysis. RT-PCR confirmed the reduced expression of AR in LNCaP cells stably expressing AR-shRNA compared to LNCaP expressing control-shRNA (inset, Fig. 2B). Data from this study further confirmed that increased sensitivity to T-cell killing by enzalutamide was dependent upon AR expression.

### Enzalutamide reduced PSA levels while improving sensitivity to PSA-specific CTL killing

**Figure 3:**
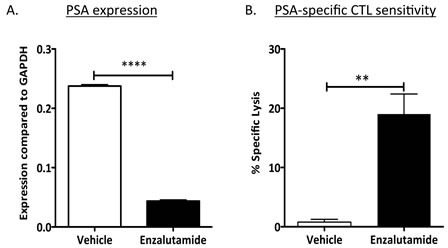
Enzalutamide mediated reduced PSA levels while improving prostate tumor-cell sensitivity to PSA-specific CD8^+^ T-cell killing (A) Expression of PSA was analyzed by RT-PCR in LNCaP (AR^+^, HLA-A2) cells treated with either vehicle (DMSO) or 10 μM enzalutamide. (B) After 48 h of either vehicle or enzalutamide treatment, cells were used as targets in a CTL lysis assay using PSA-specific CD8^+^ CTLs as effector cells at an E:T ratio of 30:1. Results are presented as mean ± S.E.M. from 3–6 replicate wells. Asterisks denote statistical significance relative to controls (**P < 0.01, ****P < 0.0001). This experiment was repeated 3–5 times with similar results.

Enzalutamide has been shown to reduce prostate-specific antigen (PSA) levels both *in vitro* and in the clinic [13, 14]. We investigated whether reduced levels of PSA would inhibit tumor sensitivity to a PSA-specific immune response in patients undergoing immunotherapy. To determine the effect of enzalutamide on PSA levels, LNCaP cells were treated *in vitro* with enzalutamide for 48 h. Real-time (RT)-PCR analysis showed a 5.5-fold reduction in levels of PSA mRNA (*P* < 0.0001) (Fig. 3A). When these cells were used as targets for PSA-specific CD8^+^ T-cell killing (Fig. 3B), treatment with enzalutamide significantly improved their sensitivity to PSA-specific T-cell killing, despite the reduction in PSA level (i.e., reduction in CTL targets) (*P* < 0.01).

### Androgen deprivation therapy modulated expression of apoptosis genes *in vitro*; enzalutamide reduced NAIP expression *in vivo*

**Figure 4:**
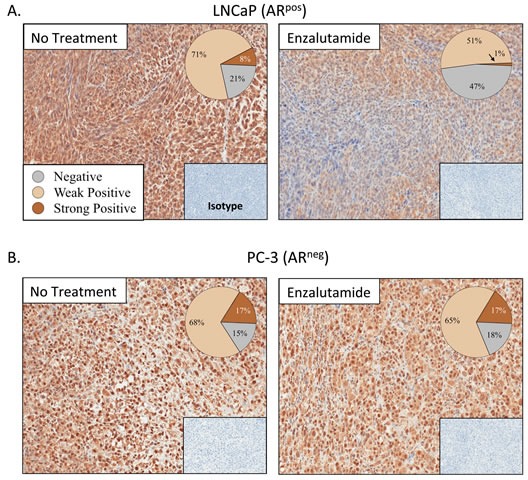
Enzalutamide reduced expression of NAIP *in vivo* Nude mice bearing LNCaP (AR^+^, HLA-A2) (A) or PC-3 (AR^−^, HLA-A24) (B) prostate xenografts were left untreated or treated with 10 mg/day of enzalutamide. After 7 days of treatment, mice were sacrificed, tumors were surgically removed, and expression of NAIP was detected by immunohistochemistry (magnification 20×) and quantified by positive pixel quantification analysis. Staining intensities are depicted in pie charts. Numbers indicate percentage of cells with negative, weak positive, or strong positive expression of NAIP. Insets are from isotype controls. This experiment was performed twice independently and similar results were obtained.

The properties of ADT that induce immunogenic modulation and sensitize prostate tumor cells to immune-mediated attack are novel and have not been previously described. We hypothesized that modulation of apoptotic genes might be part of the mechanism of action of ADT-induced CTL sensitization. We analyzed the expression of 96 genes involved in the process of apoptosis by RT-PCR in LNCaP cells treated with enzalutamide or abiraterone *in vitro*. Of these 96 genes, 3 were up-regulated and 12 were down-regulated > 2-fold by enzalutamide treatment. Abiraterone treatment resulted in a > 2-fold up-regulation of 11 genes and down-regulation of 14 genes. Further analysis showed that only 9 genes were down-regulated by both enzalutamide and abiraterone (Table 1). Among these 9 genes, one in particular, NAIP, was down-regulated 14-fold by enzalutamide and 5-fold by abiraterone treatment. To examine the reduced expression of NAIP *in vivo*, either LNCaP or PC-3 cells were transplanted into nude mice. Here, female mice were utilized to model a patient with treatment mediated castrate levels of systemic testosterone where the only source of testosterone would be from the tumor itself. Once tumors reached 500 mm^3^, mice were left untreated or treated with 10 mg/day of enzalutamide. After 7 days of treatment, tumors were subjected to immunohistochemistry staining to detect the presence and intensity of NAIP expression. NAIP showed much less intense staining in LNCaP tumors harvested from mice treated with enzalutamide (Fig. 4A, right panel) compared to LNCaP tumors harvested from untreated mice (Fig. 4A, left panel). Positive pixel analysis (insets, Fig. 4A) from 2 independent experiments demonstrated a significant 2- to 8-fold reduction in tumor cells that strongly stained for NAIP in enzalutamide-treated tumors compared to untreated tumors (*P* < 0.01). Furthermore, the overall population of enzalutamide-treated tumor cells expressing NAIP significantly decreased by 1.5-fold (*P* < 0.01) compared to untreated tumors, and there was a significant 2.2-fold increase (*P* < 0.01) in tumor area that did not express NAIP in enzalutamide-treated tumors compared to untreated tumors. In contrast, in mice harboring PC-3 tumors, treatment with enzalutamide did not mediate significant changes in NAIP expression. Similarly intense staining was seen in harvested tumors from enzalutamide-treated and untreated PC-3 tumors (Fig. 4B). Positive pixel analysis demonstrated similar NAIP expression in enzalutamide-treated and untreated PC-3 tumors (insets, Fig. 4B).

**Table 1:** Modulation of apoptosis genes in LNCaP treated with enzalutamide or abiraterone 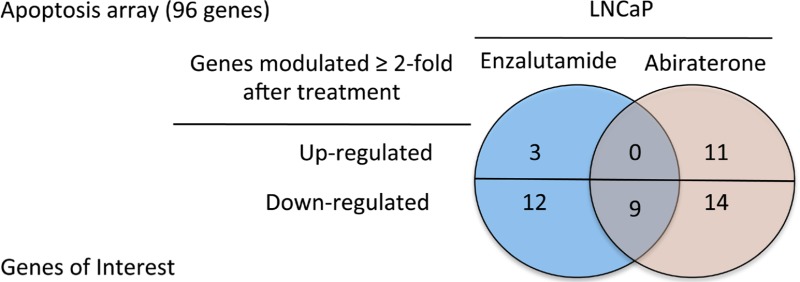

Gene	Name	Function	Enzalutamide	Abiraterone
CASP1	Caspase 1	Proapoptosis	−2.17	−4.05
CD27	CD27 molecule	Proapoptosis	−2.26	−3.50
HRK	Harakiri, BCL-2 interacting protein	Proapoptosis	−2.86	−8.57
CASP8	Caspase 8	Proapoptosis	−2.96	−2.25
CASP5	Caspase 5	Proapoptosis	−3.50	−4.22
TNFSF8	Tumor necrosis factor (ligand) super family, member 8	Proapoptosis	−3.50	−7.51
TNFRSF25 (DR3)	Tumor necrosis factor receptor super family, member 25	Proapoptosis	−3.70	−7.41
DAPK1	Death-associated protein kinase 1	Proapoptosis	−6.53	−13.73
NAIP	NLR family, apoptosis inhibitory protein	Antiapoptosis	−13.90	−4.72

LNCaP (AR^POS^) prostate tumor cells were treated with vehicle (DMSO) or 10 μM enzalutamide or abiraterone for 48 hours. Cells were harvested, RNA was extracted and apoptosis array was performed as per the manufacturer's instructions. Nine genes were modulated more than 2-fold by either enzalutamide or abiraterone treatment.

### The antiapoptotic gene NAIP is involved in the molecular mechanism of enzalutamide-mediated immunogenic modulation

**Figure 5:**
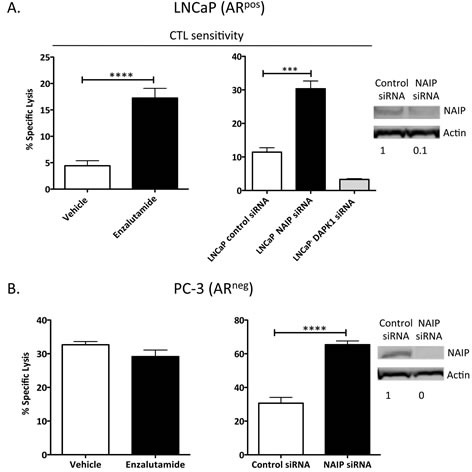
Silencing NAIP expression increased AR^+^ and AR^−^ prostate tumor cells' sensitivity to CD8^+^ T-cell killing LNCaP (AR^+^, HLA-A2) (A) and PC-3 (AR^−^, HLA-A24) (B) prostate cancer cells were treated with vehicle (DMSO) or 10 μM enzalutamide (left panels) or treated with control siRNA, NAIP siRNA, or DAPK1 siRNA (right panels) for 48 h and used as targets in a CTL lysis assay using CEA-specific or MUC1-specific CD8^+^ T cells, respectively, as effector cells at an E:T ratio of 30:1. NAIP expression after tumor cells were treated with control or NAIP siRNA was detected by western blot (insets). Results are presented as mean ± S.E.M. from 3–6 replicate wells. Asterisks denote statistical significance relative to controls (***P < 0.001, ****P < 0.0001). This experiment was repeated 3–5 times with similar results.

To investigate the role of NAIP in immunogenic modulation and subsequent improvement in CTL sensitivity mediated by enzalutamide, we transiently reduced the expression of NAIP in LNCaP cells *in vitro* using NAIP siRNA, and confirmed this reduced expression by western blot (inset, Fig. 5A). NAIP expression in LNCaP cells transfected with NAIP siRNA was reduced by 90% 48 h post-transfection, compared to LNCaP cells transfected with control siRNA. In a parallel experiment, we treated LNCaP cells with vehicle or enzalutamide for 48 h and used these cells as targets for CEA-specific CTL lysis. As shown in Figures 1A and 5A (left panel), treatment with enzalutamide, previously shown to reduce NAIP expression *in vitro* (Table 1) and *in vivo* (Fig. 4A), significantly enhanced the sensitivity of LNCaP cells to CEA-specific CD8^+^ T-cell killing (*P* < 0.0001). Similarly, reduced expression of NAIP in LNCaP cells treated with NAIP siRNA also significantly increased tumor cells' sensitivity to T cell-mediated killing (*P* < 0.001) (Fig. 5A, right panel). These data suggest that NAIP played a major role in immunogenic modulation. Besides NAIP, another gene of interest was death-associated protein kinase 1 (DAPK1), as enzalutamide down-regulated this gene 6.5-fold (Table 1). To evaluate the importance of DAPK1, we used DAPK1 siRNA to reduce the expression of DAPK1 and found that reduced expression of DAPK1 did not increase sensitivity of LNCaP cells to T-cell killing. This suggested that DAPK1 did not play a major role in enzalutamide-mediated immunogenic modulation (Fig. 5A, right panel).

To validate the importance of NAIP in the process of immunogenic modulation, PC-3 cells previously shown to be unaffected by enzalutamide (Fig. 1B) were transfected with either control or NAIP siRNA for 48 h, and reduced expression of NAIP was confirmed by western blot (inset, Fig. 5B). PC-3 cells were also independently treated with vehicle or enzalutamide. Forty-eight hours after enzalutamide or siRNA treatment, the cells were used as targets for MUC1-specific T-cell killing. As previously shown, enzalutamide did not improve the sensitivity of PC-3 cells to T-cell killing (Fig. 5B, left panel). However, reduced expression of NAIP in PC-3 cells mediated a significant improvement in T cell-mediated killing (*P* < 0.0001) (Fig. 5B, right panel).

### Enzalutamide improved the sensitivity of LNCaP/AR(cs) cells to T cell-mediated killing

**Figure 6:**
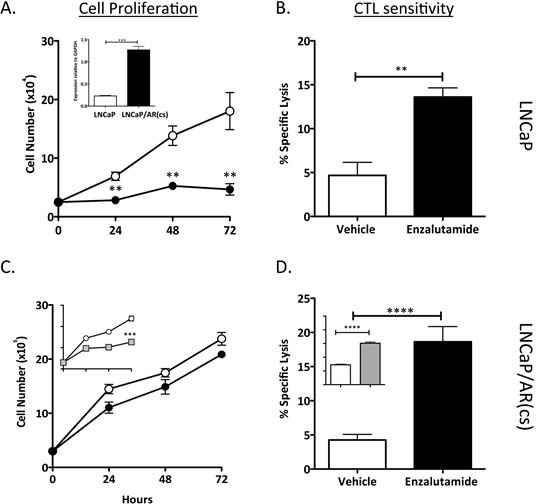
Enzalutamide improved the sensitivity of prostate tumor cells that overexpressed AR to T cell-mediated killing Overexpression of AR in the cell line LNCaP/AR(cs) was determined by RT-PCR (panel A inset). LNCaP (A) and LNCaP/AR(cs) (C) cells were treated with vehicle (DMSO; open symbols), 10 μM (closed symbols), or 30 μM enzalutamide (grey symbols). Cell proliferation was determined at indicated time points. After 48 h of either vehicle or enzalutamide treatment, LNCaP (B) and LNCaP/AR(cs) (D) cells were used as targets in a CTL lysis assay using CEA- or PSA-specific CD8^+^ T cells as effector cells at an E:T ratio of 30:1. Asterisks denote statistical significance relative to controls (**P < 0.01, ***P < 0.001, ****P < 0.0001).

CRPC is commonly associated with increased expression of AR, arising from amplification or mutation of the AR gene as well as other mechanisms [15]. Amplification of AR is arguably a major mechanism of treatment resistance in CRPC [16]. We investigated whether enzalutamide-mediated immunogenic modulation could improve the sensitivity of prostate tumor cells engineered to overexpress AR to T cell-mediated killing. RT-PCR confirmed the overexpression of AR by 5-fold in LNCaP/AR(cs) cells (inset, Fig. 6A). To determine the effect of enzalutamide on cell proliferation, LNCaP and LNCaP/AR(cs) cells were treated *in vitro* with vehicle (DMSO) or 10 μM enzalutamide (Fig. 6B). Treatment with 10 μM enzalutamide significantly inhibited the growth of LNCaP cells (*P* < 0.01) (Fig. 6A), but did not inhibit the proliferation of LNCaP/AR(cs) cells (Fig. 6C). A higher, but still clinically feasible, dose of enzalutamide (30 μM) inhibited the growth of LNCaP/AR(cs) cells by 50% while having no effect on the cells' viability (Fig. 6C, inset). To determine whether enzalutamide could increase the sensitivity of these ADT resistant cells to T-cell killing, LNCaP/AR(cs) cells were treated with 10 μM enzalutamide *in vitro* and used as target cells in either CEA- or PSA-specific CD8^+^ T cell-mediated killing assays. Exposing LNCaP (Fig. 6B) and LNCaP/AR(cs) (Fig. 6D) cells to 10 μM enzalutamide significantly improved the cells' sensitivity to T-cell killing (*P* < 0.01). Exposure of LNCaP/AR(cs) cells to 30 μM also significantly improved the cells' sensitivity to T-cell killing (*P* < 0.01). There was no significant difference in the improved CTL sensitivity by treatment of the of LNCaP/AR(cs) cells with 10 μM or 30 μM enzalutamide.

## DISCUSSION

Localized prostate cancer is treated with surgery, radiotherapy, or watchful waiting, while recurrent disease is further treated with ADT [1]. Most patients on ADT eventually develop CRPC, characterized by a rise in PSA and subsequent progression of disease despite castrate blood levels of testosterone [14]. While it was initially thought that CRPC was completely resistant to ADT, several studies have demonstrated that CRPC remains dependent on androgen signaling for growth and that CRPC is sensitive to manipulation of androgen signaling [3]. Enzalutamide and abiraterone are FDA-approved for ADT in patients with metastatic CRPC. Both enzalutamide and abiraterone have been evaluated in numerous clinical trials that resulted in improved overall survival in patients with metastatic CRPC [4-6, 9].

Immunogenic modulation describes a cascade of phenotypic and molecular events occurring when conventional therapies alter the phenotype of tumor cells, rendering them more susceptible to immune-mediated attack [9]. The molecular mechanisms of immunogenic modulation include a) changes in the surface phenotype of cancer cells, including exposure of calreticulin on the outer leaflet of the plasma membrane, b) down-regulation of antiapoptotic and/or prosurvival genes, and c) modulation of components of the APM [17-23]. The numerous immunomodulatory effects of standard therapies can be exploited to enhance the antitumor activity induced by immunotherapy. Immunogenic modulation by enzalutamide was first described in murine prostate carcinomas [10], where enzalutamide up-regulated MHC-I and Fas on the surface of tumor cells, thus improving the cells' sensitivity to T-cell killing. In those studies, treatment with enzalutamide did not alter the number or function of T cells. Enzalutamide-mediated immunogenic modulation increased the efficacy of a therapeutic cancer vaccine in TRAMP mice with spontaneous prostate tumors, which subsequently translated to significant improvements in overall survival [10].

Here, we investigated whether ADT, particularly with enzalutamide, could induce immunogenic modulation in human prostate carcinomas. Our findings demonstrated that ADT with enzalutamide or abiraterone mediated immunogenic modulation that rendered human prostate tumor cells more sensitive to T-cell killing compared to treatment with vehicle (Fig. 1). Immunogenic modulation by ADT was dependent upon AR expression, since improved tumor-cell sensitivity to T cells was only seen in AR^+^ prostate cancer cells (Figs. 1 and 2). In addition, we were able to show that despite mediating a reduction in PSA levels (Fig. 3A), enzalutamide still improved prostate tumor cells' sensitivity to PSA-specific T-cell killing (Fig. 3B). This is particularly important because enzalutamide is currently being tested in combination with PROSTVAC, a cancer vaccine composed of a series of poxviral vectors engineered to express PSA and a triad of human T-cell costimulatory molecules [24]. Evaluation of immune response from patients treated with PROSTVAC showed that 57% of patients had a ≥ 2-fold increase in PSA-specific T cells [25]. Furthermore, patients who mounted the greatest increase in PSA-specific T cells (≥ 6-fold) had improved overall survival compared with patients who did not mount as great an increase in PSA-specific T cells [26]. Our data suggest that an enzalutamide-mediated reduction in PSA level (i.e., reduction in CTL target) would not reduce the sensitivity of prostate tumor cells to PSA-specific immunity generated by the host. However, *in vitro* treatment with enzalutamide did not increase the sensitivity of prostate tumor cells to subsequent radiation therapy (Suppl. Fig. 1).

The mechanism by which ADT, particularly with enzalutamide, induces immunogenic modulation in human prostate carcinomas appears to be distinct from the molecular mechanism in murine cells. In murine TRAMP-C2 cells, treatment with enzalutamide *in vitro* up-regulated MHC-I and Fas [10]. Treatment with enzalutamide induced a modest up-regulation of tumor antigens and cell-surface molecules in AR-expressing LNCaP human prostate carcinomas (Suppl. Table 1). Up-regulation of cell-surface expression of APM and calreticulin has also been described as a molecular mechanism of immunogenic modulation [17], although exposing LNCaP cells to enzalutamide *in vitro* did not induce significant changes in expression of APM and calreticulin (Suppl. Table 2). Treatment with either enzalutamide or abiraterone *in vitro*, however, mediated major changes in several apoptotic genes in LNCaP cells. In particular, NAIP was markedly down-regulated in LNCaP cells treated *in vitro* with enzalutamide (14-fold) or abiraterone (5-fold) (Table 1). NAIP is a member of a family of inhibitors of apoptosis proteins (IAPs) [27] that directly inhibit cell death by inhibiting activated caspases. IAPs may be overexpressed in a variety of malignancies and may contribute to apoptosis resistance and drug resistance, as well as tumor progression [28]. The main function of NAIP is to protect motor neurons in the spinal cord from apoptosis. Deficiency of NAIP is associated with the most severe types of spinal muscular atrophy, a hereditary neurodegenerative disorder [29]. Emerging evidence suggests that increased expression of IAPs, particularly NAIP, plays a role in the development of drug- and hormone-resistant prostate cancer [30-36]. The exact mechanism of NAIP involvement in prostate cancer progression has not been determined. In addition, some evidence suggests a link between nuclear factor-κB (NF-κB) activity and NAIP expression [30], and that castration increases levels of NAIP mRNA and NF-κB DNA-binding activity.

Increasing evidence suggests that modulation of apoptotic genes may play a major role in sensitizing tumor cells to T cell-mediated killing [23]. It was previously shown that modulation of the Bcl-2 family of apoptosis proteins increased the sensitivity of head and neck squamous cell carcinoma to T cell-mediated killing. Here, we found that treatment with enzalutamide or abiraterone reduced the expression of a different apoptosis protein, NAIP, *in vitro* (Table 1). To confirm the reduced expression of NAIP *in vivo*, nude mice were implanted with LNCaP or PC-3 tumors and treated for 7 days with 10 mg/day of enzalutamide, a dose equivalent to a therapeutic dose in humans [4, 10]. Immunohistochemistry analysis of NAIP expression on LNCaP tumors harvested from enzalutamide-treated mice confirmed a significant reduction in NAIP expression post-enzalutamide treatment compared to untreated tumors (Fig. 4A). The reduction of NAIP was not seen in AR^−^ PC-3 prostate tumors (Fig. 4B), further confirming that immunogenic modulation by enzalutamide is strictly dependent upon AR expression (Figs. 1 and 2). The functional importance of NAIP in immunogenic modulation was confirmed by demonstrating that a reduction in NAIP, whether mediated by treatment with enzalutamide or NAIP siRNA, in either AR^+^ or AR^−^ prostate cancer cells, improved the cells' sensitivity to T cell-mediated killing (Fig. 5). These novel findings suggest the importance of NAIP in sensitizing prostate cancer cells to immune-mediated attack and warrant further studies in other carcinomas.

A majority of prostate cancer patients treated with ADT will eventually progress [37]. Several mechanisms of resistance contribute to CRPC, such as AR amplification, AR mutations, AR splice variants, post-translational modifications of AR, AR-coregulators and collaborating factors, and AR transcriptional activity [37]. We used LNCaP/AR(cs) cells engineered to overexpress AR by 5-fold over parental LNCaP cells to identify the major mechanism of immunogenic modulation. We were thus able to show that enzalutamide, although it did not affect the proliferation of LNCaP/AR(cs) cells, did mediate immunogenic modulation, rendering the cells more susceptible to T-cell killing despite increased AR expression levels. FDA approval of Provenge [38], along with encouraging clinical results for the therapeutic vaccine PROSTVAC [39] and immune checkpoint inhibitors such as PDL-1 and CTLA-4 [24, 40], represent significant milestones in the field of immunotherapy. Findings from this study now suggest that enzalutamide could potentially be used as an immunomodulatory agent in combination with these immunotherapeutic agents, even in patients who have developed treatment resistance.

To our knowledge, we report here for the first time a) the properties of ADT with enzalutamide and abiraterone that induce immunogenic modulation and render human prostate carcinomas more sensitive to immune-mediated attack; b) that the properties of ADT that induce immunogenic modulation are strictly dependent on AR expression; c) the molecular mechanism of enzalutamide-mediated immunogenic modulation in human prostate cancer cells is modulation of the expression of the antiapoptotic gene NAIP; d) the functional importance of NAIP in rendering human prostate cancer cells sensitive to immune-mediated killing; and e) that enzalutamide improves the sensitivity of prostate cancer cells harboring AR amplification, the major mechanism of ADT resistance, to T cell-mediated killing.

In reported clinical trials, more than 30% of patients did not respond to enzalutamide and continued to have rising PSA levels (5, 19). Moreover, nearly all patients who initially respond to ADT will ultimately develop ADT resistance [37]. Findings from this study provide a further rationale for combination strategies that include ADT, particularly with enzalutamide, and immunotherapy as a promising treatment option for prostate cancer, and especially for patients who have had minimal to modest responses to enzalutamide or have developed ADT resistance. A clinically active therapeutic vaccine such as PROSTVAC-VF is an attractive option in combination with enzalutamide for treatment of CRPC. In a phase II trial of PROSTVAC-VF in metastatic CRPC patients (*n* = 125), the vaccine was well tolerated and was associated with a 44% reduction in death rate and an 8.5-month improvement in median overall survival compared to placebo [39]. Clinical trials evaluating the efficacy of PROSTAC-VF plus enzalutamide in CRPC [41] and in nonmetastatic castration-sensitive prostate cancer [42] are currently underway.

## MATERIALS AND METHODS

### Tumor cells

LNCaP (HLA-A2, AR^+^) and PC-3 (HLA-A24, AR^−^) prostate adenocarcinoma cells were purchased from American Type Culture Collection (Manassas, VA) and maintained in the recommended medium. LNCaP cells stably expressing control or AR shRNA were generously provided by Dr. Paul Rennie (Vancouver Prostate Centre, Vancouver, BC). The stably transfected LNCaP cells were maintained as previously described [12]. LNCaP cells stably overexpressing AR (LNCaP/AR(cs)) were a gift from Dr. Charles Sawyers (Memorial Sloan-Kettering Cancer Center, New York, NY) and were maintained in the recommended medium with 10% FBS [43].

### Tumor-cell proliferation

To evaluate the effect of enzalutamide or abiraterone on cell proliferation, LNCaP or PC-3 prostate cancer cells were treated *in vitro* with vehicle (DMSO) or 10 μM enzalutamide or abiraterone (Selleckchem, Houston, TX) in complete media. Cells were harvested 24, 48, or 72 h after exposure, and the total number of adherent viable cells was determined by trypan blue exclusion. Viability was confirmed by 7AAD staining.

### CD8^+^ CTL lines

HLA-A2-restricted CEA-specific CTLs recognize the CEA peptide epitope YLSGANLNL (CAP-1) [44-46]. HLA-A2-restricted PSA-specific CTLs recognize the PSA peptide epitope VLSNDVCAQV [46]. The MUC1-specific CTL lines recognize the MUC1 peptide epitope ALWGQDVTSV (HLA-A2-restricted) or KYHPMSEYAL (HLA-A24-restricted) [47].

### Cytotoxicity assays

To determine T cell-mediated killing, cytotoxicity assays were performed as previously described [10]. Tumor cells were treated with vehicle or 10 μM enzalutamide or abiraterone. At the indicated times, cells were harvested and counted. Equal numbers of effector target cells from all treatment were plated with respective cytotoxic T cells. The E:T ratio was held at 30:1. and adherent cells were used as targets in a standard cytotoxicity assay using indium-111 (GE Health Care, Vienna, VA).

### RNA isolation, quantitative real-time PCR and apoptosis array

Total RNA was isolated from tumor-cell lines using the RNeasy Extraction Kit (Qiagen, Valencia, CA) and reverse-transcribed into cDNA using the Advantage RT-for-PCR Kit (Clontech, Mountain View, CA). cDNA (2.5–10 ng) was used in quantitative RT-PCR reactions using probes specific for AR (Hs00901571_m1), PSA (Hs02576345_m1), and GAPDH (4326317E). Relative mRNA expression levels of 96 genes involved in apoptosis were assessed using an apoptosis PCR array (SA Biosciences, Valencia, CA) as per the manufacturer's instructions. RT-PCR was performed on the 7300 Real-Time PCR System (Applied Biosystems, Carlsbad, CA). Where indicated, values were calculated as expression relative to GAPDH, as previously described [48].

### Enzalutamide diet

For use in an animal model, enzalutamide, which is administered orally to humans, was formulated into rodent diet. Enzalutamide admixed with Research Standard Diet (Research Diets, Inc., New Brunswick, NJ) was fed to animals at a dose level equivalent to the therapeutic level for humans, previously determined to be 10 mg/day [4, 10].

### Immunohistochemistry

All mice were housed and maintained in microisolator cages under specific pathogen-free conditions and in accordance with the guidelines of the Association for Assessment and Accreditation of Laboratory Animal Care. All experimental studies were carried out with the approval of the NIH Intramural Animal Care and Use Committee. Nude mice (Charles River, Wilmington, MA) were implanted s.c. in the right flank with 5 × 10^6^ LNCaP or PC-3 prostate tumor cells. When tumors reached a volume of 500 mm^3^, mice were either left untreated or treated with 10 mg/day of enzalutamide for 7 days. Mice were then sacrificed and tumors were surgically removed, fixed, and prepared in paraffin sections. NAIP expression was detected via immunohistochemistry using a rabbit polyclonal antibody to NAIP (Novus Bio, Littleton, CO) according to the manufacturer's instructions. Entire slides were digitally scanned by an Aperio ScanScope CS system and analyzed by Aperio ImageScope Viewer software (Aperio Technologies Inc., Vista, CA). Statistical analysis was performed using 3–7 murine tumors, each prepared as a complete stained tumor section. Positive tumor regions were determined using the Positive Pixel Count v9 algorithm. Negative controls included omission of primary antibody with PBS and matched rabbit isotype antibody. In all cases, necrotic areas of tumor were excluded from analysis.

### RNA interference (siRNA)

siRNA duplexes targeting NAIP sequences and control were purchased from Origene (Rockville, MD). LNCaP or PC-3 cells were transfected with NAIP siRNA or control siRNA according to the manufacturer's instructions. The interference of NAIP expression was confirmed by RT-PCR using TaqMan probes for NAIP (Hs03037952_m1, Applied Biosystems) or western blot.

### Western blot

For protein blotting, tumor cells were lysed in RIPA buffer modified with 1 mM PMSF (Cell Signaling Technology, Beverly, MA). Proteins (20–40 μg) were resolved using 4%–12% Tris-glycine SDS-PAGE (Life Technologies, Carlsbad, CA) and transferred to nitrocellulose membranes. Primary antibodies specific for AR and NAIP were acquired from Abcam (Cambridge, MA). Blots were incubated with antirabbit IRDye secondary antibodies (LI-COR Biotechnology, Lincoln, NE), and detected using the Odyssey Infrared Imaging System (LI-COR Biotechnology). Intensity of individual bands was quantified using ImageJ software (NIH, Bethesda, MD).

### Statistical analysis

Significant differences between multiple treatment groups were determined by unpaired Student's *t* test with a 2-tailed distribution, unless otherwise indicated, and Disclosure of Potential Conflicts of Interest.

## SUPPLEMENTARY MATERIAL, FIGURES AND TABLES


