# Supplementary material for: Androgen deprivation therapy sensitizes prostate cancer cells to T-cell killing through androgen receptor dependent modulation of the apoptotic pathway
Source: Oncotarget. 2014 Sep 3;5(19):9335–48. doi: 10.18632/oncotarget.2429 (PMC4253438; doi:10.18632/oncotarget.2429)
Supplement: Supplementary file 1 [file oncotarget-05-9335-s001.pdf]

Androgen deprivation therapy sensitizes prostate cancer cells to T-cell killing through androgen receptor dependent modulation of the apoptotic pathway

Supplementary Material

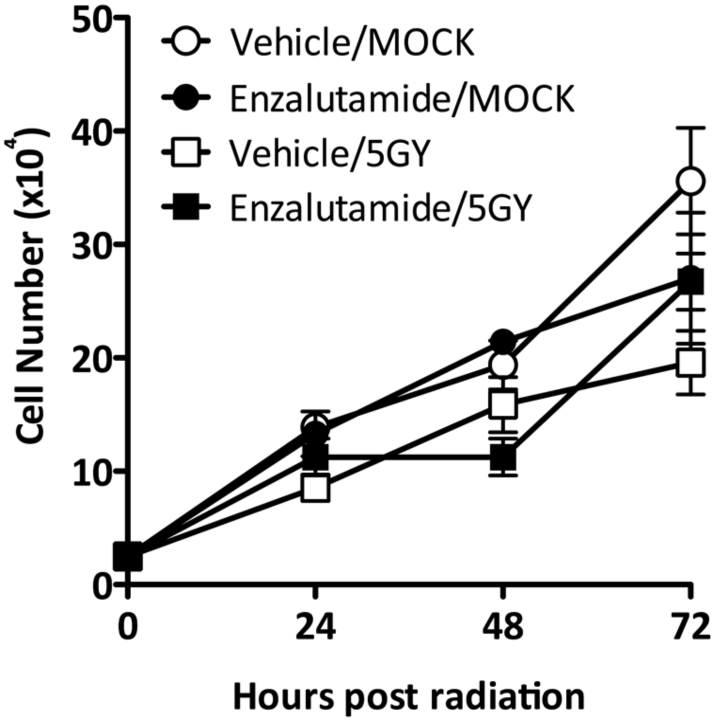

Supplemental Figure 1. Enzalutamide did not enhance the sensitivity of prostate tumor cells to subsequent radiation treatment. LNCaP cells were treated with vehicle (DMSO; open circles or open squares) or 10µM enzalutamide (closed circles or closed squares) for 72h. At the end of enzalutamide treatment, cells were washed and subjected to mock irradiation (circles) or 5GY irradiation (squares). Cells were washed and plated, and cell proliferation was determined at indicated time points. Results are presented as mean ± S.E.M. from 3–6 replicate wells. This experiment was repeated 3–5 times with similar results.

[Enzalutamide] (μM)		% Positive cells (MFI)					
		**Fas**	**ICAM-1**	**CEA**	**MUC-1**	**MHC-I**	**Calreticulin**
**LNCaP**	0	19.5 (566)	8.2 (353)	5 (258)	15 (550)	11 (404)	11.6 (339)
	10	13.7 (**652**)	9.2 (359)	5.6 (221)	15.2 (460)	14 (405)	9.5 (321)
**PC-3**	0	4 (448)	6.1 (1028)	0.3 (2649)	2.2(2290)	0.2 (12677)	6.8 (757)
	10	**7.3 (1079**)	**9.8** (1186)	**2.1** (1730)	**6.9** (698)	1.2 (3555)	**10.7** (830)

Supplemental Table 1. Enzalutamide increases the expression of cell surface markers and/or tumor antigens. Human prostate tumor cells were exposed to vehicle (0μM) or 10μM enzalutamide for 48h and levels of Fas, ICAM-1, MHC-1, CEA, MUC-1 and calreticulin at the cell surface were measured by flow cytometry. Isotype control antibody staining was ≤ than 5%. Numbers indicate the percentage of positive cells and values in bracket indicate mean fluorescent intensity (MFI). Bold indicates marked upregulation (≥ 20% increase in percent of cells and/or MFI).

	% Positive (MFI)	
	**Vehicle**	**Enzalutamide**
LMP2	82.7% (108)	87.6% (107)
LMP7	< 1%	**12.9**% (29)
LMP10	71.8% (70)	81.3% (59)
TAP1	34.3% (26)	25.8% (22)
TAP2	83.8% (109)	85.6% (88)
Calnexin	82.7% (127)	87.6% (**93**)
Calreticulin	39.4% (29)	**5.4**% (25)
Tapasin	48.7% (38)	52.1% (34)

Supplemental Table 2. Effect of enzalutamide on protein expression of antigen processing machinery (APM) components in prostate carcinoma cells. Human prostate (LNCaP) carcinoma cells were exposed to enzalutamide (10 μM) or vehicle (DMSO) control. After 72 h, cells were analyzed by flow cytometry for intracellular expression of indicated APM components. Numbers indicate percentage of positive cells. Numbers in parentheses denote mean fluorescent intensity (MFI). Bold denotes significant modulation (≥ 30% change in percent of cells or MFI not observed in isotype control vs. untreated cells).
